# Supplementary material for: Effects of dietary supplementation with Lactobacillus acidophilus on the performance, intestinal physical barrier function, and the expression of NOD-like receptors in weaned piglets
Source: PeerJ. 2018 Dec 18;6:e6060. doi: 10.7717/peerj.6060 (PMC6302781; doi:10.7717/peerj.6060)
Supplement: Supplemental Information 2 [file peerj-06-6060-s002.docx]

The protein abundance of TJs in the intestinal tissues of weaned piglets

| Occludin | Control | Treatment | *P* value |
| --- | --- | --- | --- |
| Jejunum | 1.00±0.03 | 1.52±0.06 | *P*<0.001 |
| Ileum | 1.00±0.02 | 1.33±0.05 | *P*<0.001 |

| ZO-1 | Control | Treatment | *P* value |
| --- | --- | --- | --- |
| Jejunum | 1.00±0.04 | 1.07±0.03 | *P*=0.087 |
| Ileum | 1.00±0.05 | 1.04±0.03 | *P*=0.333 |
