# Supplementary material for: Effects of dietary supplementation with Lactobacillus acidophilus on the performance, intestinal physical barrier function, and the expression of NOD-like receptors in weaned piglets
Source: PeerJ. 2018 Dec 18;6:e6060. doi: 10.7717/peerj.6060 (PMC6302781; doi:10.7717/peerj.6060)
Supplement: Supplemental Information 3 [file peerj-06-6060-s003.docx]

The relative mRNA expression of NLR‐signaling-pathway‐related genes in the intestinal tissues of weaned piglets.

| NOD1 | Control | Treatment | *P* value |
| --- | --- | --- | --- |
| Jejunum | 1.00±0.02 | 0.33±0.03 | *P* <0.001 |
| Ileum | 1.00±0.06 | 0.98±0.04 | *P*=0.603 |

| RIPK2 | Control | Treatment | *P* value |
| --- | --- | --- | --- |
| Jejunum | 1.00±0.05 | 0.86±0.04 | *P*=0.020 |
| Ileum | 1.00±0.04 | 0.97±0.04 | *P*=0.374 |

| NF‐κB | Control | Treatment | *P* value |
| --- | --- | --- | --- |
| Jejunum | 1.00±0.07 | 0.56±0.03 | *P*=0.001 |
| Ileum | 1.00±0.07 | 0.92±0.04 | *P*=0.184 |

| NLRP3 | Control | Treatment | *P* value |
| --- | --- | --- | --- |
| Jejunum | 1.00±0.06 | 0.70±0.02 | *P*=0.001 |
| Ileum | 1.00±0.03 | 0.85±0.06 | *P*=0.025 |

| caspase‐1 | Control | Treatment | *P* value |
| --- | --- | --- | --- |
| Jejunum | 1.00±0.04 | 0.78±0.05 | *P*=0.003 |
| Ileum | 1.00±0.05 | 0.80±0.03 | *P*=0.003 |

| IL‐1β | Control | Treatment | *P* value |
| --- | --- | --- | --- |
| Jejunum | 1.00±0.07 | 0.78±0.04 | *P*=0.011 |
| Ileum | 1.00±0.04 | 0.88±0.03 | *P*=0.019 |

| IL‐18 | Control | Treatment | *P* value |
| --- | --- | --- | --- |
| Jejunum | 1.00±0.05 | 0.81±0.03 | *P*=0.004 |
| Ileum | 1.00±0.05 | 0.87±0.03 | *P*=0.015 |
