# Supplementary figures and images for: Effects of dietary supplementation with Lactobacillus acidophilus on the performance, intestinal physical barrier function, and the expression of NOD-like receptors in weaned piglets
Source: PeerJ. 2018 Dec 18;6:e6060. doi: 10.7717/peerj.6060 (PMC6302781; doi:10.7717/peerj.6060)

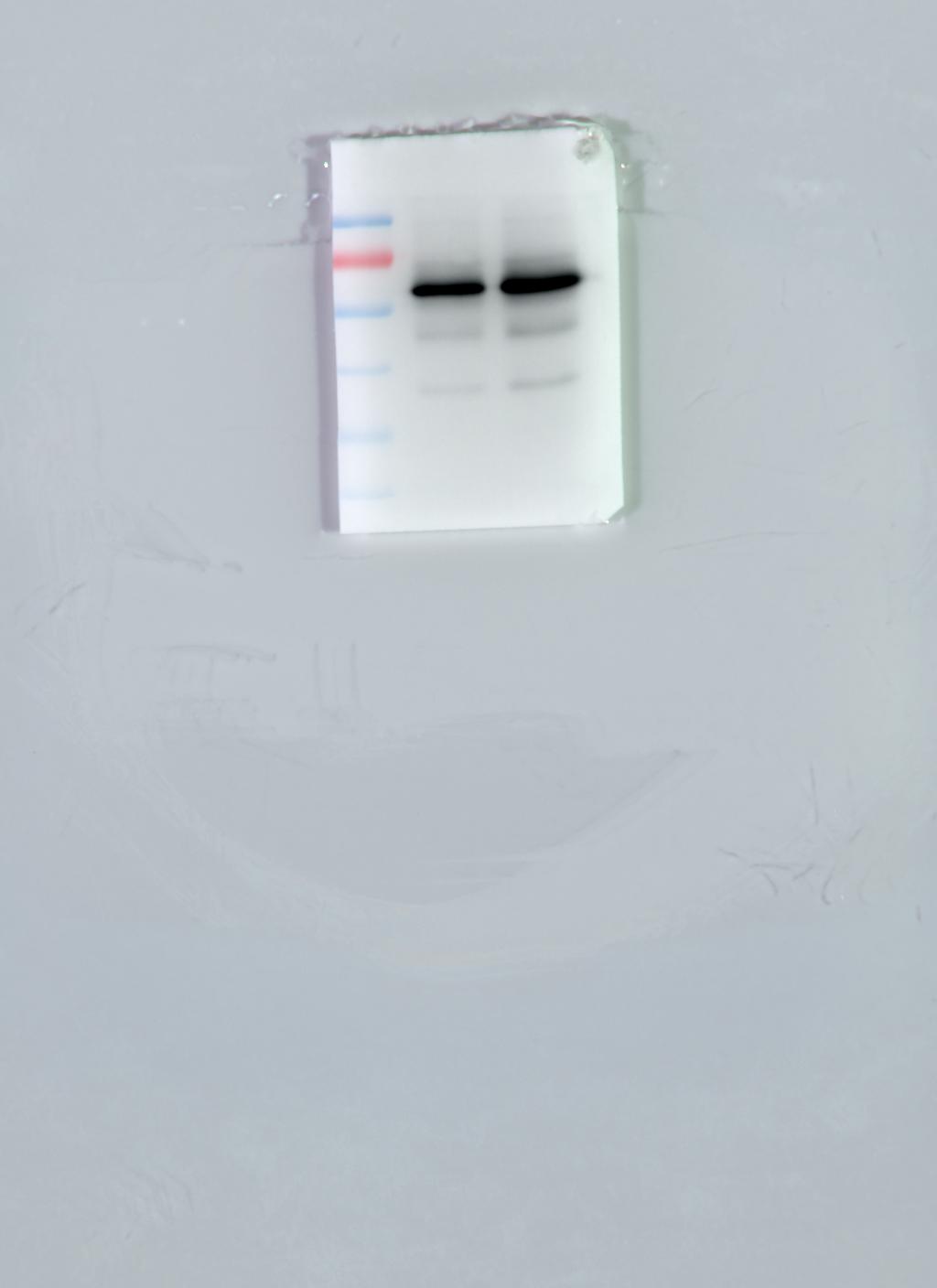

Supplement: Supplemental Information 4 — The protein abundance of occludin in the jejunum tissue of weaned piglets. [file peerj-06-6060-s004.jpg]

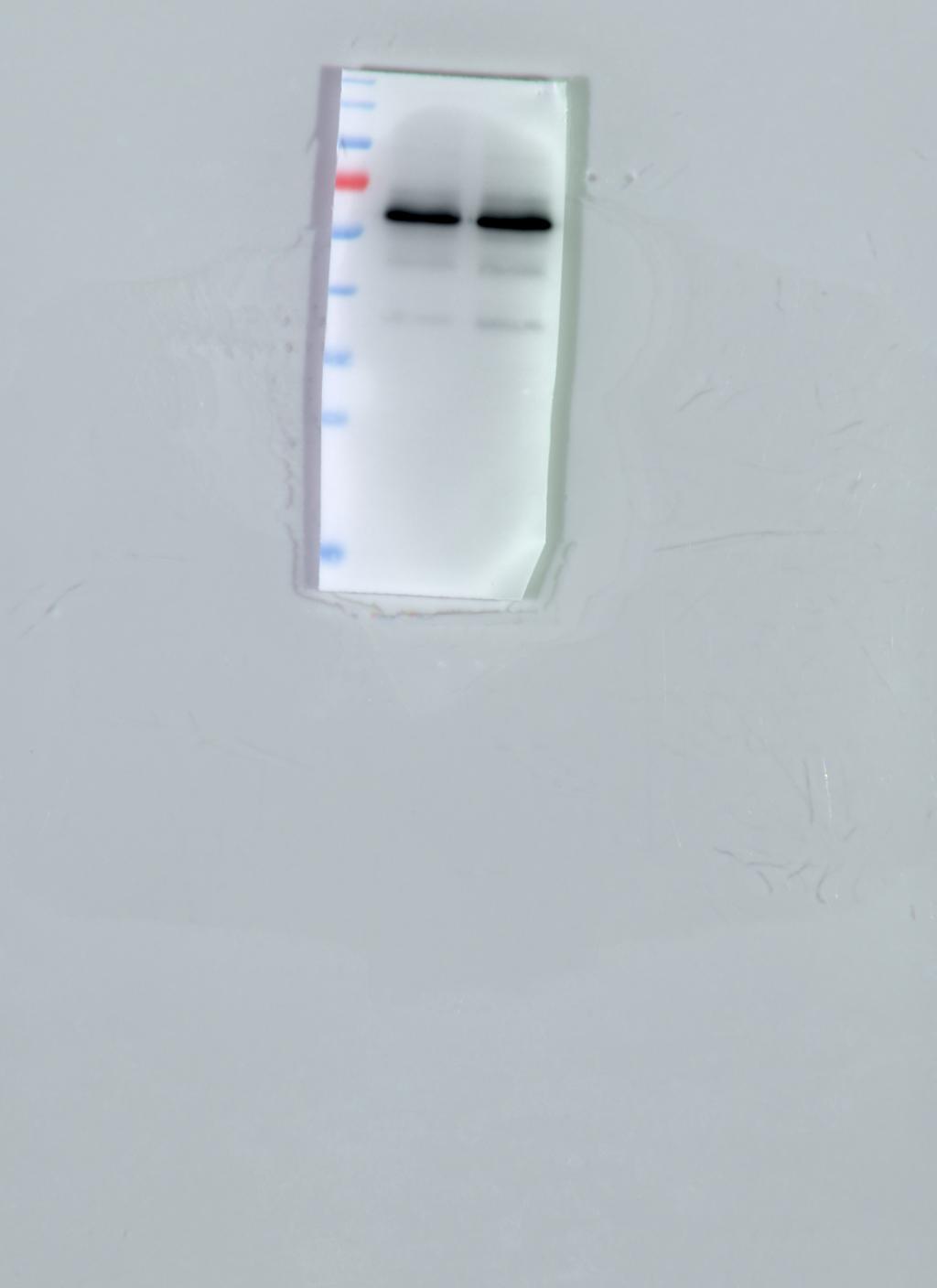

Supplement: Supplemental Information 5 [file peerj-06-6060-s005.jpg]

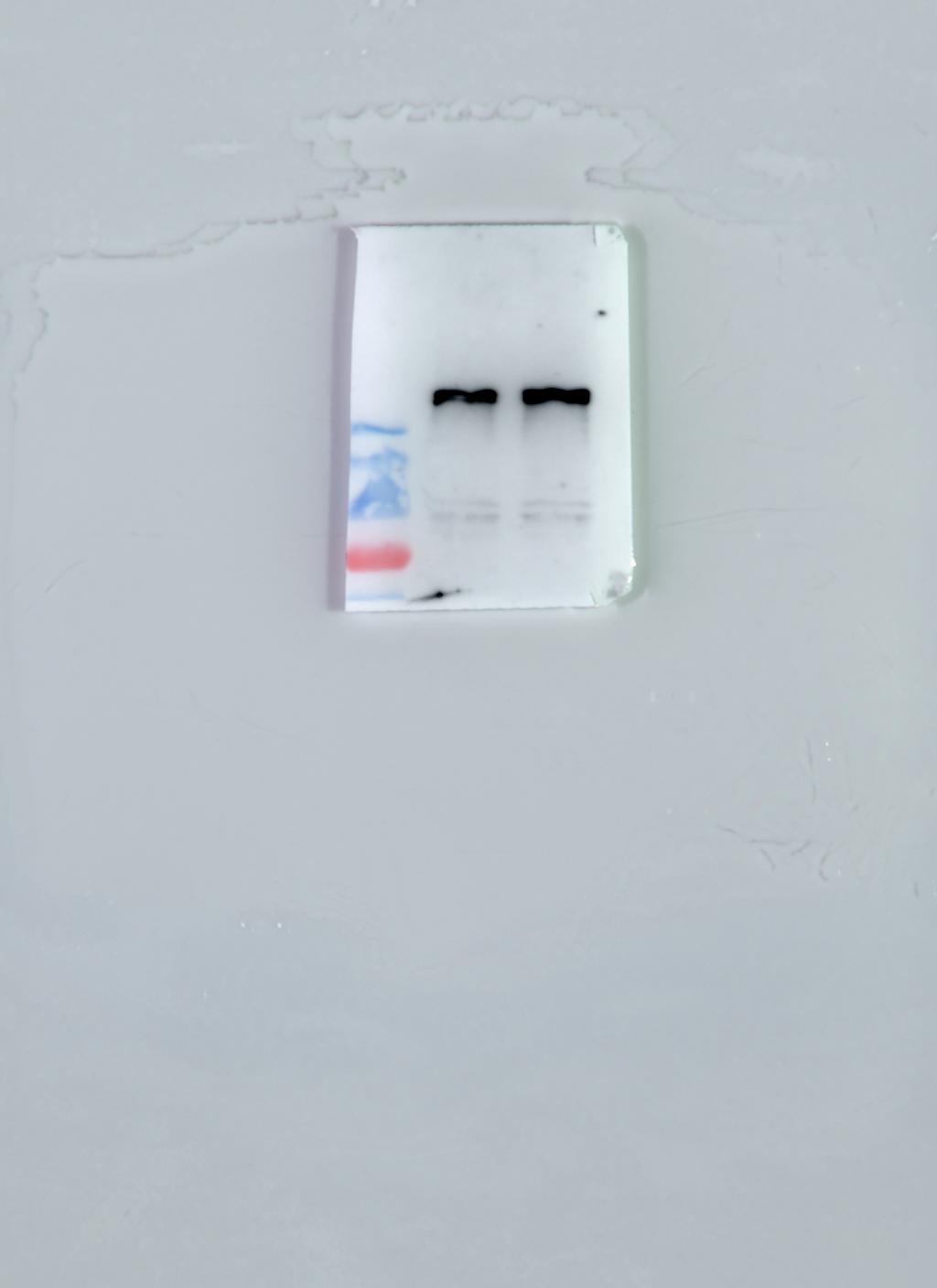

Supplement: Supplemental Information 6 [file peerj-06-6060-s006.jpg]

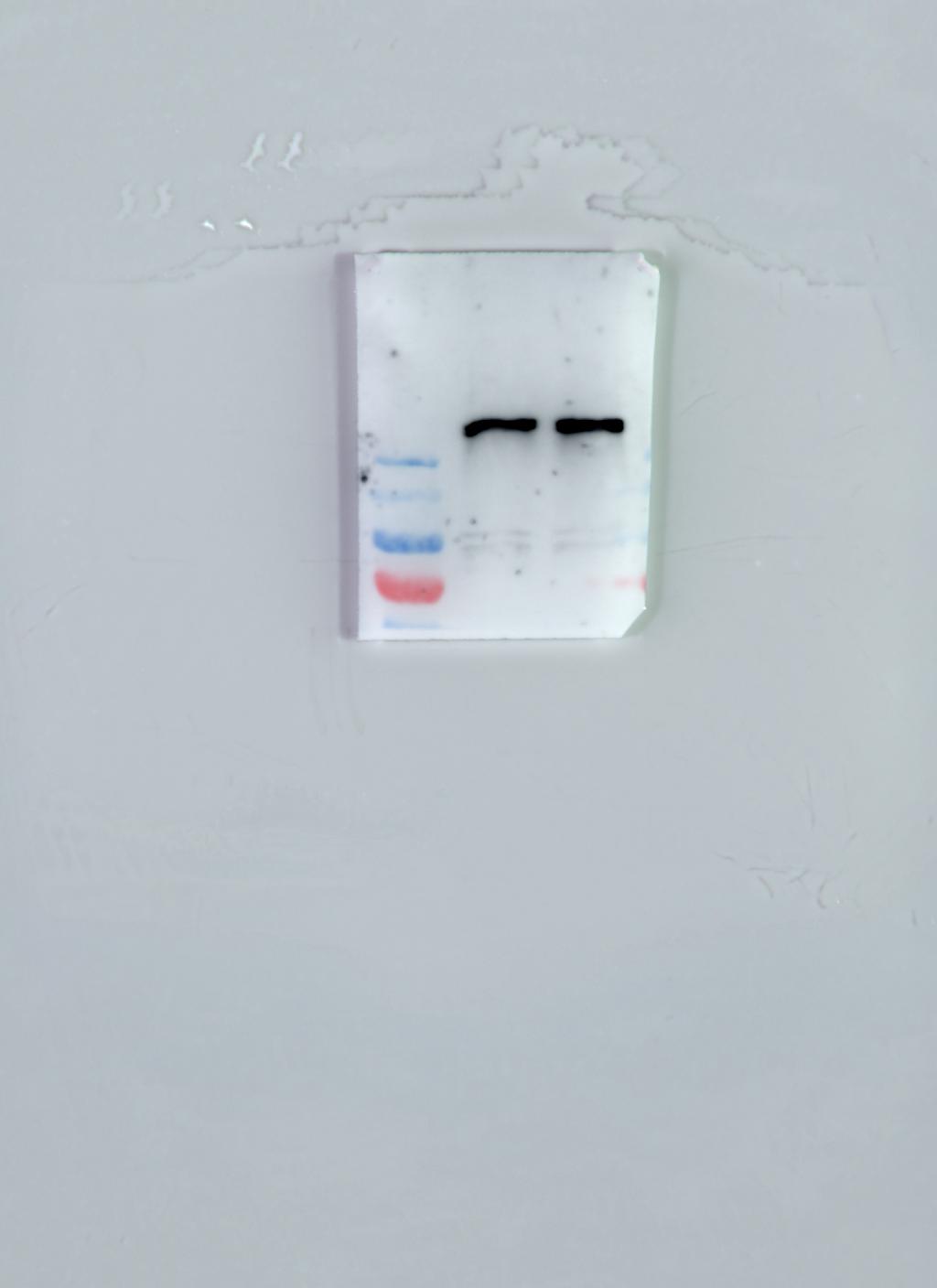

Supplement: Supplemental Information 7 [file peerj-06-6060-s007.jpg]

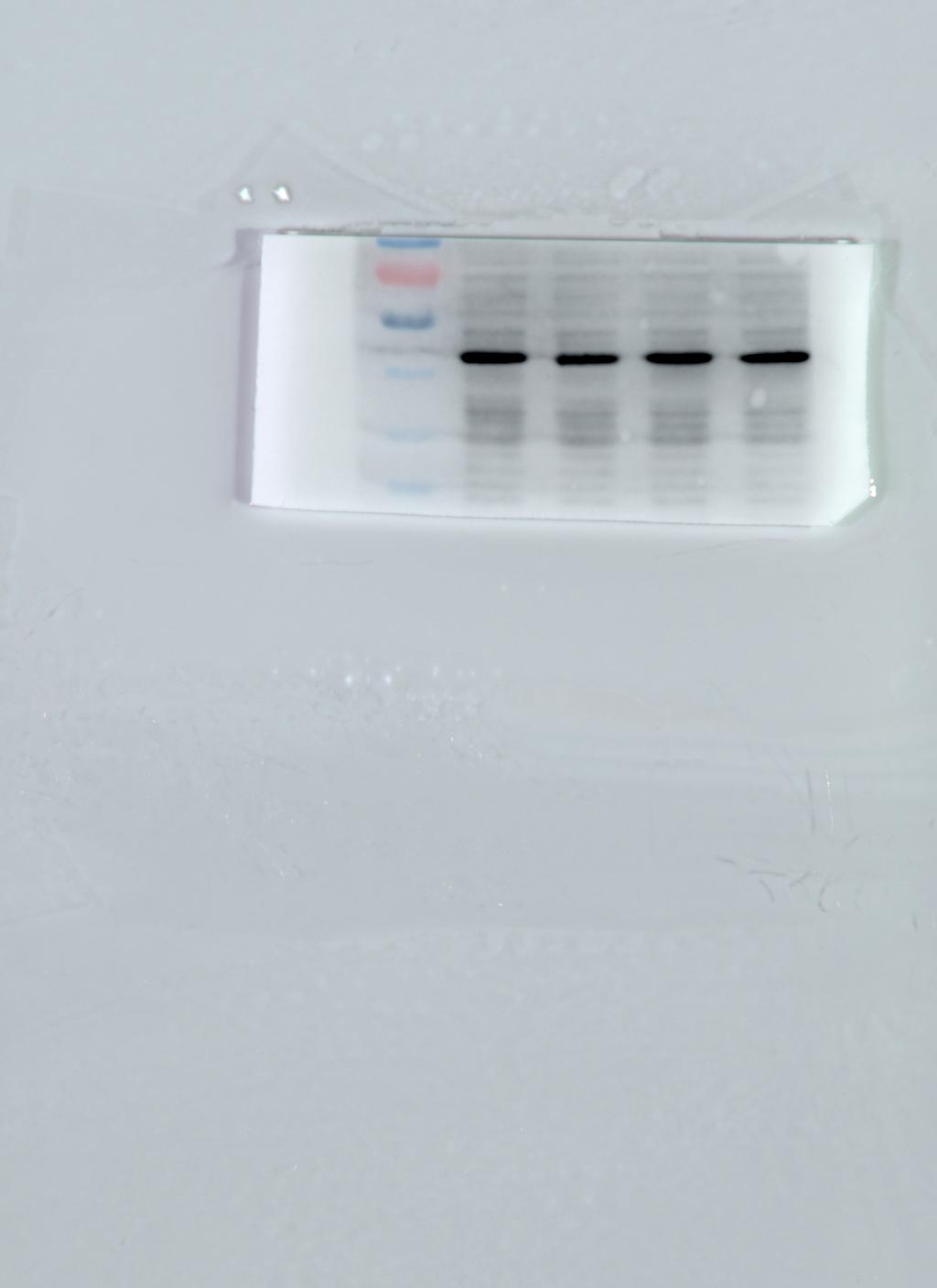

Supplement: Supplemental Information 8 [file peerj-06-6060-s008.jpg]
